# Tendon lengthening and fascia release for healing and preventing diabetic foot ulcers: a systematic review and meta-analysis

**DOI:** 10.1186/s13047-015-0085-6

**Published:** 2015-07-30

**Authors:** Sarah M. Dallimore, Michelle R. Kaminski

**Affiliations:** Eastern Health Podiatry Department, Maroondah Hospital, Davey Drive, Ringwood East, VIC 3135 Australia; Eastern Health Podiatry Department, Angliss Hospital, Albert Street, Upper Ferntree Gully, VIC 3156 Australia

**Keywords:** Achilles tendon lengthening, Diabetic foot ulcer, Gastrocnemius recession, Plantar fascia release

## Abstract

**Background:**

Diabetic foot ulcers have a devastating impact on an individual’s health-related quality of life and functional status. Additionally, diabetic foot ulcers impose a significant economic burden on our health care systems as a result of complications such as infection, hospitalisation and amputation. The current gold standard treatment for diabetic foot ulcers is total contact casting. However, the rate of ulcer recurrence is high, indicating the need for more effective long-term treatment options. Therefore, the aim of this study was to systematically identify, critique and evaluate all literature investigating the effectiveness of Achilles tendon lengthening, gastrocnemius recession and selective plantar fascia release in healing and preventing diabetic foot ulcers.

**Review:**

Searches were conducted in MEDLINE, CINAHL, AMED, EMBASE and The Cochrane Library from the earliest available date to November 2014. Methodological quality of included studies was assessed using the Downs and Black checklist. Data from randomised-controlled trials were analysed using random effects meta-analysis. For all other studies, data were analysed descriptively.

Eleven studies (614 participants) were included in the review, with a median sample size of 29 participants. Meta-analysis of two randomised-controlled trials found that there was no statistically significant difference between Achilles tendon lengthening or gastrocnemius recession and total contact casting for time to healing of diabetic foot ulcers (mean difference, MD, 8.22 days; 95 % CI, −18.99 to 35.43; *P* = 0.55; *I*^*2*^ = 34 %) and the rate of ulcers healed (risk ratio, RR, 1.06; 95 % CI, 0.94 to 1.20; *P* = 0.34; *I*^*2*^ = 41 %). The rate of ulcer recurrence was significantly lower following Achilles tendon lengthening or gastrocnemius recession than total contact casting (RR, 0.45; 95 % CI, 0.28 to 0.72; *P* < 0.001; *I*^*2*^ = 0 %).

**Conclusions:**

Achilles tendon lengthening and gastrocnemius recession appear to be effective surgical treatments for healing diabetic foot ulcers. The rate of ulcer recurrence was lower following Achilles tendon lengthening or gastrocnemius recession procedures compared to total contact casting treatment alone. Therefore, these surgical procedures may provide viable treatment options for the management and prevention of diabetic foot ulcers. Further rigorous randomised-controlled trials with longer follow-up are required to determine the long-term effectiveness and safety of these procedures.

**Electronic supplementary material:**

The online version of this article (doi:10.1186/s13047-015-0085-6) contains supplementary material, which is available to authorized users.

## Background

Diabetic foot ulceration is a global public health problem. Foot ulcers have a devastating impact on an individual’s health-related quality of life and functional status. Further complications such as infection, hospitalisation and amputation are common. An estimated 15 % of people with diabetes will develop an ulcer during their lifetime [1] and approximately 85 % of all non-traumatic lower extremity amputations in people with diabetes are preceded by a foot ulcer [1, 2]. Consequently, foot ulcers impose a significant economic burden on our health care systems [1, 3].

Sensory neuropathy and high plantar pressures have been implicated in the development of diabetic foot ulceration [3, 4]. The current gold standard treatment is total contact casting (TCC), which is a conservative treatment method shown to be effective in reducing plantar pressures [5, 6] and healing neuropathic ulcers in people with diabetes [7–13]. However, reported rates of ulcer recurrence are high, ranging from 19 % to 81 % [9, 10, 12–15]. These high recurrence rates may be explained by the temporary offloading effect provided by TCC. It may also reflect the difficulty in preventing ulcer recurrence in neuropathic feet through means of footwear, insoles and appropriate foot care, and patient compliance with these modalities [14]. Thus, a more effective long-term treatment option for preventing the development and recurrence of diabetic foot ulceration is needed.

Limited ankle joint dorsiflexion (i.e., equinus deformity) is associated with elevated plantar pressures, which subsequently increases the risk of plantar ulceration in people with diabetes [16, 17]. There is a threefold risk of equinus deformity in those with diabetes compared to those without [16]. This is thought to be a result of non-enzymatic glycosylation [4, 18, 19], which alters the structure and function of collagen within connective tissues, such as tendons and fascia, causing stiffness. Shortening of the Achilles tendon can result in plantarflexion at the ankle and increased plantar forefoot pressures during gait [17]. Achilles tendon lengthening (ATL) and gastrocnemius recession (GR) procedures have been found to increase ankle joint dorsiflexion [15, 20, 21] and ATL has been found to reduce plantar forefoot pressures [20]. Hence, these procedures may provide viable options for the management of diabetic foot ulcers.

However, potential risks associated with these surgical procedures must also be considered. One such risk is over-lengthening of the tendon which can result in a calcaneal gait, chronic heel ulceration and tendon rupture. With this in mind, selective plantar fascia release (SPFR) has been proposed as an alternative procedure to ATL for the management of diabetic foot ulcers [22].

To our knowledge, there has been no systematic review and meta-analysis investigating the effectiveness of these surgical procedures in the management of diabetic foot ulcers. Therefore, the aim of this review was to systematically identify, critique and evaluate all literature investigating the effectiveness of ATL, GR and SPFR in healing and preventing diabetic foot ulcers. The primary outcome measures were time to healing of the ulcer, rate of ulcers healed, rate of ulcer recurrence, and rate of transfer ulcers (i.e., a new ulcer). The secondary outcome measures were complications and adverse events.

## Review

### Methods

#### Registration

This systematic review was prospectively registered on PROSPERO (Registration No. CRD42013006290).

#### Search strategy

Searches were conducted from the earliest available date to September 2013 in MEDLINE, CINAHL, AMED, EMBASE and The Cochrane Library. The detailed search strategy used for MEDLINE is available in Additional file 1. The reference lists of included articles were checked and citation tracking using Google Scholar was performed to identify any further relevant citations. Searches were repeated in November 2014 to ensure any new citations were identified and assessed for eligibility prior to submission.

#### Selection criteria

The titles and abstracts of records identified in the search were independently screened by two reviewers (SMD and MRK) based on the inclusion and exclusion criteria (Table 1). Full text was obtained for any articles in which a decision to include or exclude the article could not be made based on the information provided in the title or abstract. Any disagreements were discussed until a consensus was reached.

**Table 1:** Inclusion and exclusion criteria

	Inclusion criteria	Exclusion criteria
Population	Studies of participants diagnosed with:	Studies in which the data was not separated for participants with and without diabetes
	i. Type 1 or Type 2 Diabetes Mellitus	
	And	
	ii. Plantar forefoot or midfoot ulceration	
Intervention	Studies of participants who have undergone one of the following procedures:	
	i. Achilles tendon lengthening	
	ii. Gastrocnemius recession	
	iii. Plantar fascia release	
Outcomes	Studies investigating:	
	i. Time to healing of the ulcer	
	And/ Or	
	ii. Rate of ulcers healed	
	And/ Or	
	iii. Rate of ulcer recurrence	
	And/ Or	
	iv. Rate of transfer (new) ulcers	
Study design	Peer-reviewed publications	Conference presentations
		Case studies
		Studies published in a language other than English

For relevant studies where the data were not separated for participants with and without diabetes, or where participants had undergone different surgical procedures, the authors were contacted for their raw data. Studies in which the raw data were obtained from responding authors were subsequently included in the review.

#### Data extraction

One author (SMD) extracted primary study data using a customised data extraction form which included information regarding study design, participant characteristics, intervention details, outcomes of interest, outcome results, complications and length of follow-up. Data extraction was independently confirmed by the second author (MRK).

#### Quality assessment

The methodological quality of included studies was assessed using the Downs and Black (1998) checklist, which has been shown to be valid and reliable [23]. It is comprised of 27 items (10 relating to reporting, three relating to external validity, seven relating to internal validity – bias, six relating to internal validity – confounding, and one relating to power) with a maximum achievable score of 32. Scoring for item 27 relating to study power was modified in this review for easier application and analysis by the assessors. Studies in which a power analysis was performed and were adequately powered were given a score of five. Studies in which a power analysis was performed but where sample sizes were borderline, or studies that were able to determine a statistically significant difference despite questionable sample sizes, were given a score of three. Studies in which a power analysis was not performed and were clearly underpowered were given a score of zero. While modification altered the total achievable score for this individual item (i.e., studies were not able to score a one, two or four), all studies were rated using the same scale and therefore comparability between the studies was not affected. Quality assessment was independently performed by two assessors (SMD and MRK). Any disagreements were discussed until a consensus was reached.

#### Data analysis

All data from randomised-controlled trials (RCTs) were analysed by meta-analysis with the inverse-variance method. Time to healing of the ulcer was analysed by calculating the mean difference (MD) and 95 % confidence intervals (CIs) using a random effects model. Where data were reported as a median and range, the mean and standard deviation were estimated [24]. Rate of ulcers healed and rate of ulcer recurrence were analysed by calculating risk ratios (RRs) and 95 % CIs using a random effects model. A *P*-value of 0.05 was considered statistically significant. Heterogeneity between the studies was determined using the *I*^*2*^ statistic [25]. Meta-analyses were conducted using Review Manager (RevMan Version 5.3. Copenhagen: The Nordic Cochrane Centre, The Cochrane Collaboration, 2014).

For all other studies, data relating to time to healing of the ulcer, rate of ulcers healed and rate of ulcer recurrence were analysed descriptively. Rate of transfer ulcers and reported complications and adverse events that occurred in the intervention groups were also analysed descriptively and were calculated in terms of number of participants. The rate of transfer ulcers included heel ulcers, however the rate of heel ulcers was also analysed separately as a complication of the procedures.

### Results

**Fig. 1:**
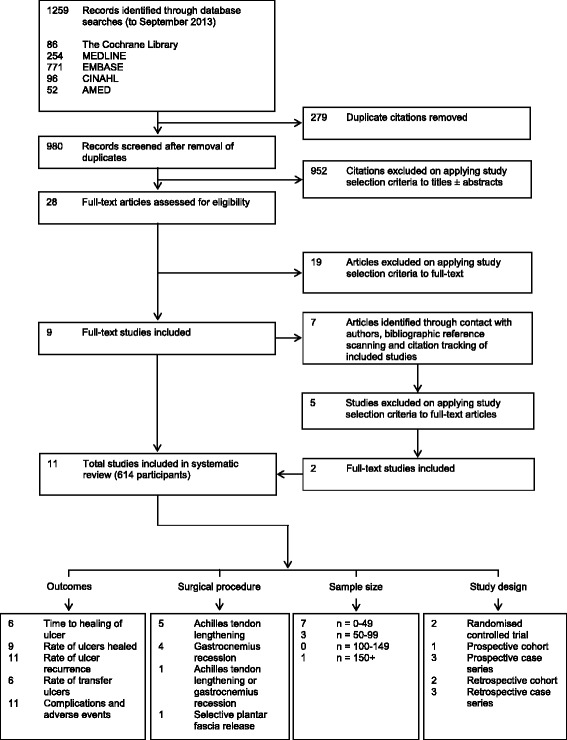
Flow diagram of search process

The results of the search process are shown in Fig. 1. A total of 11 studies (614 participants) [15, 21, 22, 26–33] were included in the review, with a median sample size of 29 participants. There were two RCTs [15, 21], one prospective cohort study [28], three prospective case series [22, 26, 30], two retrospective cohort studies [27, 33] and three retrospective case series [29, 31, 32]. Characteristics of included studies are presented in Table 2. A detailed table of the extracted data (including individual study results and complications) is available in Additional file 2.

**Table 2:** Characteristics of included studies

Study	Number of participants / Number of ulcers		Baseline age (years)^a^	Male (%)	Duration of diabetes (years)^b^	Duration of ulcer (months)^c^	Ulcer location	
	Intervention group	Control group					Forefoot	Midfoot
Allam, 2006* [21]	15	14	55.0 ± 11.0	Not reported	20.0 ± 11.0	Median = 42.0 (range 10–72)	●	
Batista *et al.*, 2011^†^ [26]	52	N/A	Mean = 66.4	Not reported	>5 years (100% participants)	N/A	●	
Colen *et al.*, 2013^‡^ [27]	138 / 145	149 / 179	58.5 ± 10.0	59 %	Not reported	Not reported	●	●
Dayer & Assal, 2009^§^ [28]	24	N/A	56.3 ± 12.4	42 %	Not reported	15.9 ± 7.6	●	
Hamilton *et al.*, 2005^¶^ [29]	7	N/A	51.3 ± 10.9	Not reported	Not reported	Not reported	●	
Kim *et al.*, 2012^†^ [22]	60 / 64	N/A	54.1 ± 14.3	62 %	>10 years (70% participants)	5.5 ± 3.6	●	
Laborde, 2005^†^ [30]	17 / 20	N/A	58.7 ± 12.3	53 %	Not reported	12.9 ± 19.3	●	
Laborde, 2009^¶^ [31]	10 / 10	N/A	60.1 ± 15.1	60 %	Not reported	16.2 ± 21.7		●
La Fontaine *et al.*, 2008^¶^ [32]	28	N/A	Median = 51.0 (range 24–72)	71 %	Not reported	Not reported	●	●
Lin *et al.*, 1996^‡^ [33]	15	21	Intervention group 45.8 ± 16.3	Intervention group 73 %	Intervention group 10.0 ± 4.4	Intervention group 11.5 ± 3.7	●	
			Control group 50.7 ± 11.8	Control group 48 %	Control group 11.7 ± 5.3	Control group 6.5 ± 3.1		
Mueller *et al.*, 2003* [15]	31	33	56.0 ± 10.0	77 %	18.4 ± 11.7	Not reported	●	
Study	Procedure			Primary outcomes				Follow-up (months)^d^
	Achilles tendon lengthening	Gastrocnemius recession	Selective plantar fascia release	Time to healing	Rate of ulcers healed	Rate of ulcer recurrence	Rate of transfer ulcers	
Allam, 2006* [21]	○	○		♦	♦	♦		Mean = 24.0
Batista *et al.*, 2011^†^ [26]	○					♦		Mean = 24.0
Colen *et al.*, 2013^‡^ [27]	○					♦	♦	35.3 ± 11.0
Dayer & Assal, 2009^§^ [28]		○		♦	♦	♦	♦	39.2 ± 12.2
Hamilton *et al.*, 2005^¶^ [29]		○		♦	♦	♦	♦	17.1 ± 7.3
Kim *et al.*, 2012^†^ [22]			○		♦	♦		Mean = 23.5
Laborde, 2005^†^ [30]		○			♦	♦	♦	34.6 ± 19.3
Laborde, 2009^¶^ [31]		○			♦	♦	♦	35.2 ± 18.5
La Fontaine *et al.*, 2008 ^¶^[32]	○			♦	♦	♦	♦	Mean = 28.8
Lin *et al.*, 1996^‡^ [33]	○			♦	♦	♦		*Intervention group* Mean = 17.3
								*Control group* Mean = 12.8
Mueller *et al.*, 2003* [15]	○			♦	♦	♦		25.2 ± 8.4

^a^Unless shown otherwise, baseline age given as mean ± standard deviation

^b^Unless shown otherwise, duration of diabetes given as mean ± standard deviation

^c^Unless shown otherwise, duration of ulcer given as mean ± standard deviation

^d^Unless shown otherwise, follow-up given as mean ± standard deviation

^*^Randomised-controlled trial

^†^Prospective case series

^‡^Retrospective cohort

^§^Prospective cohort

^¶^Retrospective case series

#### Quality assessment

The quality assessment scores of the 11 studies ranged from nine to 27 with a mean and standard deviation of 17 ± 5. The results of quality assessment are shown in Table 3. A table outlining the detailed scoring for each of the 27 items is available in Additional file 3.

**Table 3:** Quality assessment scores

Reference	Reporting (max = 11)	External validity (max = 3)	Internal validity		Power (max = 5)	Total (max = 32)
			Bias (max = 7)	Confounding (max = 6)		
Allam, 2006 [21]	9	0	4	2	3	18
Batista *et al.*, 2011 [26]	5	0	2	2	0	9
Colen *et al.*, 2013 [27]	9	2	5	2	3	21
Dayer *et al.*, 2009 [28]	10	0	3	2	0	15
Hamilton *et al.*, 2005 [29]	9	2	2	3	0	16
Kim *et al.*, 2012 [22]	9	0	4	2	3	18
Laborde, 2005 [30]	9	2	2	2	0	15
Laborde, 2009 [31]	8	2	2	2	0	14
La Fontaine *et al.*, 2008 [32]	9	3	4	3	5	24
Lin *et al.*, 1996 [33]	8	0	3	1	0	12
Mueller *et al.*, 2003 [15]	11	0	5	6	5	27

Ten studies [15, 21, 22, 27–33] scored eight or higher for reporting, indicating the majority of included studies provided sufficient information regarding study objectives, methods and results. More than half of the studies [15, 21, 22, 26, 28, 33] scored zero for external validity, demonstrating studies had suboptimal recruitment methods or failed to adequately describe them. Eight studies [21, 22, 26, 28–31, 33] scored six or less (out of a maximum 13) for internal validity, which assessed bias in subject selection and measurement of outcomes. Six studies [26, 28–31, 33] scored zero for power, indicating they had insufficient power to detect a statistically significant effect.

#### Time to healing of the ulcer

**Fig. 2:**
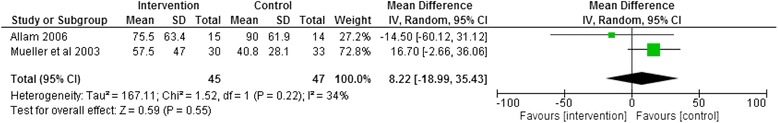
Forest plot of studies investigating time (days) to healing of the ulcer for Achilles tendon lengthening or gastrocnemius recession versus total contact casting

Two RCTs [15, 21] investigated the effectiveness of ATL or GR (intervention group) versus TCC (control group) on time to healing of diabetic foot ulcers. Participants in the intervention group of one study [21] had either ATL or GR, which was determined based on dorsiflexion measurement at the ankle joint with the knee straight and flexed. A meta-analysis found that there was no statistically significant difference in mean time to healing of diabetic foot ulcers between the intervention and control groups (MD, 8.22 days; 95 % CI, −18.99 to 35.43; *P* = 0.55; *I*^*2*^ = 34 %) (Fig. 2). The mean time to healing reported in the intervention groups was 57.5 days [15] and 75.5 days [21]. As the results were not separated for the ATL and GR participants in the study by Allam (2006) [21], comparisons between the two procedures cannot be made. Two other studies [32, 33] found a mean time to healing following ATL of 65.8 days and 39.3 days respectively.

#### Rate of ulcers healed

**Fig. 3:**
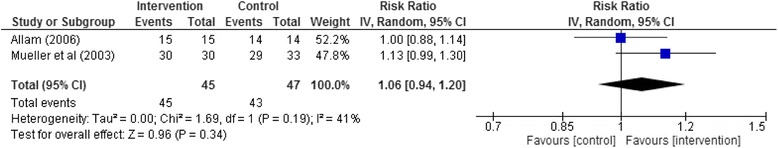
Forest plot of studies investigating the rate of ulcers healed for Achilles tendon lengthening or gastrocnemius recession versus total contact casting

Two RCTs [15, 21] investigated the rate of ulcers healed following ATL or GR (intervention group) versus TCC (control group). A meta-analysis found that there was no statistically significant difference in the rate of ulcers healed between the intervention and control groups (RR, 1.06; 95 % CI, 0.94 to 1.20; *P* = 0.34; *I*^*2*^ = 41 %) (Fig. 3). The reported rate of ulcers healed in the intervention groups was 100 %. Three other studies [31–33] found that the rate of ulcers healed following ATL or GR was 88.9 %, 85.7 % and 93.3 % respectively. One study [22] investigated the rate of ulcers healed following SPFR, which was found to be 56.3 %.

#### Rate of ulcer recurrence

**Fig. 4:**
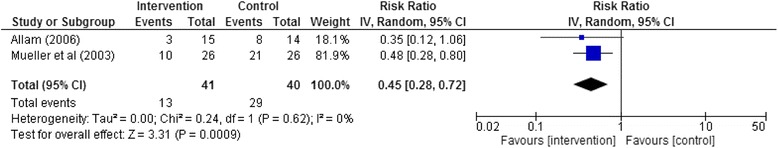
Forest plot of studies investigating the rate of ulcer recurrence for Achilles tendon lengthening or gastrocnemius recession versus total contact casting

Two RCTs [15, 21] investigated the rate of ulcer recurrence following ATL or GR (intervention group) versus TCC (control group). A meta-analysis found that the rate of ulcer recurrence was significantly lower in the intervention group than the control group (RR, 0.45; 95 % CI, 0.28 to 0.72; *P* < 0.001; *I*^*2*^ = 0 %) (Fig. 4). The rate of ulcer recurrence reported in the intervention groups was 38.5 % [15] and 20 % [21]. In the study by Allam (2006) [21], ulcer recurrence rates for the ATL and GR groups were 16.7 % and 22.2 % respectively. Four other studies [26, 31–33] found that the rate of ulcer recurrence following ATL or GR was 7.7 %, 12.5 %, 41.7 % and 0 % respectively. One study [22] investigated the rate of ulcer recurrence following SPFR, which was found to be 0 %.

#### Rate of transfer ulcers

The rate of heel transfer ulcers reported in the intervention groups of the two RCTs was 12.9 % [15] and 20 % [21]. The results were not separated for the ATL and GR groups in the study by Allam (2006) [21]. Two other studies [31, 32] found that the rate of transfer ulcers (including heel ulcers) following ATL or GR was 0 % and 21.4 % respectively.

#### Complications and adverse events

The complications and adverse events are listed in Additional file 2. In the two RCTs [15, 21], a combined total of seven participants (15.2 %) in the intervention groups developed a heel ulcer following ATL or GR. Overall, three deaths (6.5 %) were recorded in the intervention groups during the follow-up period, however only one of these occurred during the treatment phase with the reported cause of death a myocardial infarction. Other reported complications following surgery included wound haematoma (6.5 %), calcaneal gait (4.3 %), and a ruptured Achilles tendon (4.3 %). One participant (2.2 %) developed an infection requiring debridement and amputation. Mueller et al. (2003) [15] also reported complications relating to TCC in both the intervention and control groups which included abrasions (12.9 % and 18.2 % respectively), falls (6.5 % and 0 % respectively) and intolerance (0 % and 9.1 % respectively). In the study by Allam (2006) [21], early complications from surgery were reported separately for the ATL and GR groups, which included wound haematoma (16.7 % and 22.2 % respectively), calcaneal gait (16.7 % and 0 % respectively), and a ruptured Achilles tendon (16.7 % and 11.1 % respectively).

Four other studies [26, 31–33] also reported complications following ATL or GR. Five heel ulcers (4.8 %) were recorded across the intervention groups of these studies during the follow-up period. There were two deaths (1.9 %), however these were reported as complications of the comorbidities of diabetes rather than the surgery [31]. One participant (1.0 %) developed an infection requiring partial foot amputation, and one participant (1.0 %) had gangrene requiring an above knee amputation. The study investigating SPFR [22] reported no complications associated with the procedure.

### Discussion

Increased plantar pressures in conjunction with neuropathy have been implicated in the development of plantar foot ulceration in people with diabetes. At present, the gold standard treatment for diabetic foot ulcers is TCC. However, ulcers often recur following cessation of this treatment, which may be explained by the temporary nature of offloading provided by TCC. Achilles tendon lengthening and GR procedures increase the range of dorsiflexion at the ankle joint, while SPFR increases the range of motion at the metatarsophalangeal joints. In theory, improved dorsiflexion range of motion at the ankle and metatarsophalangeal joints decreases plantar forefoot pressures and the risk of plantar foot ulceration.

This review found that ATL and GR appear to be effective surgical treatments for healing diabetic foot ulcers when an equinus deformity is present. Interestingly, there was no statistically significant difference between these procedures and the current gold standard treatment of TCC for time to healing of the ulcer and the rate of ulcers healed. However, the rate of ulcer recurrence was found to be lower in participants who had undergone ATL or GR procedures compared to those treated with TCC alone. One RCT [15] included in this review also found that re-ulceration occurred significantly earlier in those managed with TCC alone compared to those who underwent ATL in conjunction with TCC (*P* = 0.03).

Conversely, surgery can expose patients to greater complications and adverse events. Our review found that the development of transfer ulcers, particularly under the heel, were the most common complications following ATL or GR procedures. Transfer ulcers may occur due to pressure being transferred elsewhere under the foot as a result of changes to foot function and/or overcorrection. As SPFR does not affect ankle joint range of motion, it may reduce the risk of heel ulcers. However, future RCTs are required to determine this. The rate of other complications in the included studies was low, which is in agreement with a previous study that found there is low morbidity associated with gastrocnemius recession [34]. At present, ATL appears to be the procedure of choice as it is relatively quick and easy to perform [35]. Further high-quality RCTs are needed to determine which of these methods may be associated with fewer complications and improved patient outcomes. Consideration of where the limited joint range of motion is present will also guide which procedure is most appropriate.

While the results of this review suggest ATL and GR may provide viable treatment options for healing and preventing diabetic foot ulcers, the long-term effectiveness and safety of these procedures remains unknown. There is also limited evidence regarding the precise mechanisms by which these procedures heal and prevent ulceration. Armstrong et al. (1999) [20] found that peak plantar forefoot pressures were reduced and ankle joint dorsiflexion was increased following ATL at eight weeks follow-up. Similarly, Mueller et al. (2003) [15] found that peak plantar forefoot pressures were reduced and ankle joint dorsiflexion was increased post-operatively following ATL. However, plantar pressures returned to baseline values at seven months follow-up. Despite this, the observed increase in ankle joint dorsiflexion remained and re-ulceration rates were significantly lower in those who had undergone ATL compared to those in the TCC group. Allam (2006) [21] also recorded a significant improvement in ankle joint dorsiflexion post ATL or GR. This decreased after two years follow-up though remained within the normal range.

Orendurff et al. (2006) [36] found that despite a statistically significant relationship between equinus deformity of the ankle and increased peak plantar forefoot pressures during walking, the relationship was weak (R^2^ = 0.149). While this may still be of clinical relevance, this result suggests that other contributing factors may also play a role (e.g., bony deformities, thickness of plantar tissues, modification of gait) [36]. Orendurff et al. (2006) [36] discuss that a prolonged period of increased pressure under the forefoot during gait, as a result of equinus deformity, may play a role in ulcer development and requires further investigation.

This is the first systematic review and meta-analysis to investigate the effectiveness of ATL, GR and SPFR in healing and preventing diabetic foot ulcers. The strengths of this review include a rigorous search strategy, studies were selected, reviewed and assessed systematically by two independent reviewers using standardised methods, the relevance of the results to clinical practice and research prioritisation were discussed, and finally, the manuscript was reported in accordance with the PRISMA guidelines [37].

The main limitation of this review was that the majority of studies were not RCTs and ranged in the level of quality. The results of studies (i.e., those that weren’t RCTs) were reported descriptively as we believe that these data add to the overall literature on this topic and are important from a clinical perspective. However, based on quality assessment, the results of these studies should be interpreted with caution. The participants in some of the studies also had concurrent surgical procedures and therefore we cannot be certain that the outcomes were a direct result of the procedures investigated in this review. Accordingly, the findings of these studies were not reported separately within the written section of the results. However, individual study results can be obtained from Table 2 (Characteristics of included studies) and Additional file 2 (Data extraction table). As there was only one study investigating SPFR, results were unable to be pooled in a meta-analysis. Furthermore, the study was not a RCT, and therefore the findings should be interpreted with caution. A further limitation was the exclusion of studies where data were not separated for participants with and without diabetes (i.e., where the raw data could not be obtained from the authors), and studies printed in a language other than English. As a result, this review may not have included all available data possible for use in meta-analyses.

## Conclusions

This review found that ATL and GR appear to be effective surgical treatments in healing diabetic foot ulcers when an equinus deformity is present. Meta-analysis found that there was no statistically significant difference between these procedures and the current gold standard treatment of TCC for time to healing of the ulcer and the rate of ulcers healed. The rate of ulcer recurrence was lower following ATL or GR procedures compared to TCC treatment alone. Therefore, these surgical procedures may provide viable treatment options for the management and prevention of diabetic foot ulcers. Further rigorous RCTs with longer follow-up are required to determine the long-term effectiveness and safety of these procedures.

## Additional files

Additional file 1:
**Search strategy for MEDLINE.** The database search strategy used for MEDLINE. (PDF 7 kb) Additional file 2:
**Data extraction table.** A table presenting the data extracted, including author and year of publication, study design, participant characteristics, details of intervention and control groups, outcome results, complications and length of follow-up. (PDF 86 kb) Additional file 3:
**Quality assessment scores for individual checklist items.** A table showing the quality assessment scores for the individual checklist items for each included study. (PDF 44 kb)

## Abbreviations

ATL: Achilles tendon lengthening
GR: Gastrocnemius recession
SPFR: Selective plantar fascia release
TCC: Total contact casting
MD: Mean difference
RR: Risk ratio
RCT: Randomised-controlled trial
