# Supplementary material for: Tendon lengthening and fascia release for healing and preventing diabetic foot ulcers: a systematic review and meta-analysis
Source: J Foot Ankle Res. 2015 Jul 30;8:33. doi: 10.1186/s13047-015-0085-6 (PMC4546251; doi:10.1186/s13047-015-0085-6)
Supplement: Additional file 1: — Search strategy for MEDLINE. The database search strategy used for MEDLINE. (PDF 7 kb) [file 13047_2015_85_MOESM1_ESM.pdf]

Additional file 1

Search strategy for MEDLINE (Search platform OvidSP; 2/9/13)

1. exp. Diabetes Mellitus
2. diabet*
3. 1 or 2
4. exp. Foot ulcer
5. ulcer*
6. wound*
7. 4 or 5 or 6
8. exp. Lower Extremity
9. exp. Foot
10. feet
11. foot
12. lower limb*
13. lower extremit*
14. 8 or 9 or 10 or 11 or 12 or 13
15. exp. Muscle, Skeletal
16. exp. Tendons
17. exp. Fascia
18. Achilles
19. gastroc*
20. soleus
21. tibialis posterior
22. perone*
23. plantar fascia
24. tendo*
25. 15 or 16 or 17 or 18 or 19 or 20 or 21 or 22 or 23 or 24
26. exp. Specialities, Surgical
27. exp. Surgical Procedures, Operative
28. exp. Orthopedic Procedures
29. surg*
30. operat*
31. procedure*
32. 26 or 27 or 28 or 29 or 30 or 31
33. 3 and 7 and 14 and 25 and 32
