# Supplementary material for: Tendon lengthening and fascia release for healing and preventing diabetic foot ulcers: a systematic review and meta-analysis
Source: J Foot Ankle Res. 2015 Jul 30;8:33. doi: 10.1186/s13047-015-0085-6 (PMC4546251; doi:10.1186/s13047-015-0085-6)
Supplement: Additional file 2: — Data extraction table. A table presenting the data extracted, including author and year of publication, study design, participant characteristics, details of intervention and control groups, outcome results, complications and length of follow-up. (PDF 86 kb) [file 13047_2015_85_MOESM2_ESM.pdf]

Additional file 2

Data extraction table

Study	Design	Number of participants (N), number of ulcers	Age (years)*, male %, duration of diabetes (years)†, duration of ulcer (months)‡	Location of ulcer	Details of intervention and control groups	Outcome results§	Complications and adverse events	Follow-up (months)¶	Comments
Allam, 2006 [21]	Randomised-controlled trial	*Intervention group*<br>N = 15<br><br>*Control group*<br>N = 14	*Age*<br>55.0 ± 11.0<br><br>*Duration of diabetes*<br>20.0 ± 11.0<br><br>*Duration of ulcer*<br>Median = 42.0 (range 10-72)	Forefoot	*Intervention group*<br>GR (Tongue in groove) or<br>ATL (Percutaneous triple hemi-section)<br><br>*Control group*<br>TCC	*Intervention group*<br>1) Time to healing of ulcer Median 30 days (range 21-221 days)<br>2) Rate of ulcers healed 15/15 (100%)<br>3) Rate of ulcer recurrence 3/15 (20.0%)<br><br>*Control group*<br>1) Time to healing of ulcer Median 49 days (range 32-230 days)<br>2) Rate of ulcers healed 14/14 (100%)<br>3) Rate of ulcer recurrence 8/14 (57.1%)	*Intervention group*<br>3 wound haematomas (20.0%)<br>2 ruptured Achilles tendons (13.3%)<br>2 calcaneal gait (13.3%)<br>3 late heel ulcers (20.0%)<br><br>*Control group*<br>1 calcaneal gait (7.1%)	Mean = 24.0	Data for time to healing of ulcer is for 6 month follow-up
Batista *et al.*, 2011 [26]	Prospective case series	N = 52	*Age*<br>Mean = 66.4<br><br>*Duration of diabetes*<br>> 5 years (100% participants)	Forefoot	ATL (Modified White with three hemi-sections) + TCC	1) Rate of ulcer recurrence 4/52 (7.7%)	None reported	Mean = 24.0	

Colen *et al.*, 2013 [27]	Retrospective cohort	*Intervention group*<br>N = 138<br>145 ulcers<br><br>*Control group*<br>N = 149<br>179 ulcers	*Age*<br>58.5 ± 10.0<br><br>*Male % = 59*	Forefoot/ Midfoot	*Intervention group*<br>ATL (2-stab method or open Z-plasty)<br>+ surgical soft tissue reconstruction<br>± tarsal tunnel release<br><br>*Control group*<br>Surgical soft tissue reconstruction<br>± tarsal tunnel release	*Intervention group*<br>1) Rate of ulcer recurrence (requiring reoperation) 3/145 (2.1%)<br><br>2) Rate of transfer ulcers 6/138 (4.3%)<br><br>*Control group*<br>1) Rate of ulcer recurrence (requiring reoperation) 45/179 (25.1%)<br><br>2) Rate of transfer ulcers 21/149 (14.1%)	*Intervention group*<br>10 suture line dehiscence (7.2%)<br>17 small plantar wounds delaying weight-bearing ambulation >28 days (12.3%)<br>2 heel transfer ulcers (1.4%)<br><br>*Control group*<br>13 suture line dehiscence (9.4%)<br>18 small plantar wounds delaying weight-bearing ambulation >28 days (12.1%)	35.3 ± 11.0	Surgical soft tissue reconstruction refers to wound closure surgery including management of bony prominences and debridement
Dayer & Assal, 2009 [28]	Prospective cohort	N = 24	*Age*<br>56.3 ±12.4<br><br>*Male %* = 42<br><br>*Duration of ulcer*<br>15.9 ± 7.6	Forefoot	GR (Strayer)<br>+ Jones extensor hallucis longus transfer and flexor hallucis longus transfer<br>± peroneus longus to peroneus brevis transfer	1) Time to healing of ulcer 31.3 ± 12.2 days<br><br>2) Rate of ulcers healed 21/22 (95.5%)<br><br>3) Rate of ulcer recurrence 0/21 (0%)<br><br>4) Rate of transfer ulcers 1/22 (4.5%)	1 death from myocardial infarction (4.2%)	39.2 ± 12.2	Data only presented for subjects who received GR
Hamilton *et al.*, 2005 [29]	Retrospective case series	N = 7	*Age*<br>51.3 ± 10.9	Forefoot	GR<br>+ peroneus longus to brevis transfer<br>+ resection of the 2nd through 5th metatarsal heads<br>+ TCC	1) Time to healing of ulcer 42.6 days (range 28-102 days)<br><br>2) Rate of ulcers healed 7/7 (100%)<br><br>3) Rate of ulcer recurrence 0/7 (0%)<br><br>4) Rate of transfer ulcers 0/7 (0%)	1 dehiscence at site of gastrocnemius incision (14.3%)<br>1 sinus from antibiotic bead placed in forefoot to treat osteomyelitis (14.3%)	17.1 ± 7.3	Data only presented for subjects with diabetes<br><br>Data for time to healing of ulcer includes non-diabetic subjects (data for this outcome not separated in study)

Kim *et al.*, 2012 [22]	Prospective case series	N = 60<br>64 ulcers	*Age*<br>54.1 ± 14.3<br><br>*Male %* = 62<br><br>*Duration of diabetes*<br>> 10 years (70% participants)<br><br>*Duration of ulcer*<br>5.5 ± 3.6	Forefoot	Selective plantar fascia release	1) Rate of ulcers healed 36/64 (56.3%)<br><br>2) Rate of ulcer recurrence 0/36 (0%)	None reported	Mean = 23.5	
Laborde, 2005 [30]	Prospective case series	N = 17<br>20 ulcers	*Age*<br>58.7 ± 12.3<br><br>*Male %* = 53<br><br>*Duration of ulcer*<br>12.9 ± 19.3	Forefoot	GR (Vulpius)<br>+ toe tenotomy for ulcers on the 1st toe<br>+ peroneus longus lengthening (z-type) for 1st metatarsal ulcers<br>+ posterior tibial lengthening (intramuscular) for 5th metatarsal & cuboid ulcers	1) Rate of ulcers healed 19/20 (95.0%)<br><br>2) Rate of ulcer recurrence 3/19 (15.8%)<br><br>3) Rate of transfer ulcers 2/17 (11.8%)	1 death (5.9%)<br>1 pulmonary embolism (5.9%)<br>1 gangrene with below knee amputation (5.9%)<br>1 gangrene with above knee amputation (5.9%)<br>1 heel transfer ulcer (5.9%)	34.6 ± 19.3	Data only presented for subjects with diabetes who received GR
Laborde, 2009 [31]	Retrospective case series	N = 10<br>10 ulcers	*Age*<br>60.1 ± 15.1<br><br>*Male %* = 60<br><br>*Duration of ulcer*<br>16.2 ± 21.7	Midfoot	GR (Vulpius)	1) Rate of ulcers healed 8/9 (88.9%)<br><br>2) Rate of ulcer recurrence 1/8 (12.5%)<br><br>3) Rate of transfer ulcers 0/9 (0%)	2 deaths (20.0%)<br>I gangrene with trans-femoral amputation (10.0%)	35.2 ± 18.5	Data only presented for subjects with diabetes
La Fontaine *et al.*, 2008 [32]	Retrospective case series	N = 28	*Age*<br>Median = 51.0 (range 24-72)<br><br>*Male %* = 71	Forefoot/ Midfoot	ATL (Hoke)	1) Time to healing of ulcer 65.8 days (range 21-504 days)<br><br>2) Rate of ulcers healed 24/28 (85.7%)<br><br>3) Rate of ulcer recurrence 10/24 (41.7%)<br><br>4) Rate of transfer ulcers 6/28 (21.4%)	5 heel transfer ulcers (17.9%)	Mean = 28.8	

Lin *et al.*, 1996 [33]	Retrospective cohort	*Intervention group*<br>N = 15<br><br>*Control group*<br>N = 21	*Intervention group*<br>*Age*<br>45.8 ± 16.3<br><br>*Male %* = 73<br><br>*Duration of diabetes*<br>10.0 ± 4.4<br><br>*Duration of ulcer*<br>11.5 ± 3.7<br><br>*Control group*<br>*Age*<br>50.7 ± 11.8<br><br>*Male %* = 48<br><br>*Duration of diabetes*<br>11.7 ± 5.3<br><br>*Duration of ulcer*<br>6.5 ± 3.1	Forefoot	*Intervention group*<br>ATL (Hoke) + TCC<br><br>*Control group*<br>TCC	*Intervention group*<br>1) Time to healing of ulcer 39.3 days (range 21-90 days)<br><br>2) Rate of ulcers healed 14/15 (93.3%)<br><br>3) Rate of ulcer recurrence 0/14 (0%)<br><br>*Control group*<br>1) Time to healing of ulcer 43.5 days (range 25-62 days)<br><br>2) Rate of ulcers healed 21/21 (100%)<br><br>3) Rate of ulcer recurrence 4/21 (19.0%)	*Intervention group*<br>1 infection at the 1st metatarsal head (12 weeks after ATL procedure) with partial foot amputation (6.7%)<br><br>*Control group*<br>None reported	*Intervention group*<br>Mean = 17.3 (range 11-26)<br><br>*Control group*<br>Mean = 12.8 (range 8-23)	Time to healing for intervention group was calculated 3 weeks post ATL (from the time of reinitiating the TCC protocol)

Mueller *et al.*, 2003 [15]	Randomised-controlled trial	*Intervention group*<br>N = 31<br><br>*Control group*<br>N = 33	*Age*<br>56.0 ± 10.0<br><br>*Male %* = 77<br><br>*Duration of diabetes*<br>18.4 ± 11.7	Forefoot	*Intervention group*<br>ATL (Hoke) + TCC<br><br>*Control group*<br>TCC	*Intervention group*<br>1) Time to healing of ulcer 57.5 ± 47.0 days<br>2) Rate of ulcers healed 30/30 (100%)<br>3) Rate of ulcer recurrence 10/26 (38.5%)<br><br>*Control group*<br>1) Time to healing of ulcer 40.8 ± 28.1 days<br>2) Rate of ulcers healed 29/33 (87.9%)<br>3) Rate of ulcer recurrence 21/26 (80.8%)	*Intervention group*<br>3 deaths (1 from MI during treatment phase) (9.7%)<br>4 heel transfer ulcers (12.9%)<br>4 abrasions from the TCC (12.9%)<br>1 deep infection requiring debridement and antibiotic therapy (3.2%)<br>2 falls in the TCC (6.5%)<br><br>*Control group*<br>3 deaths (9.1%)<br>1 toe amputation (3.0%)<br>6 abrasions from the TCC (18.2%)<br>3 intolerant to cast and immobilized with removable pressure relief boot (9.1%)	25.2 ± 8.4 (range 15.6-46.8)	Average follow-up time was measured from initial healing of ulcer

[*] Unless shown otherwise, age given as mean ± standard deviation

[†] Unless shown otherwise, duration of diabetes given as mean ± standard deviation

[‡] Unless shown otherwise, duration of ulcer given as mean ± standard deviation

[§]Subjects available for follow-up

[¶]Unless shown otherwise, follow-up given as mean± standard deviation

Abbreviations: GR, gastrocnemius recession; ATL, Achilles tendon lengthening; TCC, total contact cast.
