# Supplementary material for: Tendon lengthening and fascia release for healing and preventing diabetic foot ulcers: a systematic review and meta-analysis
Source: J Foot Ankle Res. 2015 Jul 30;8:33. doi: 10.1186/s13047-015-0085-6 (PMC4546251; doi:10.1186/s13047-015-0085-6)
Supplement: Additional file 3: — Quality assessment scores for individual checklist items. A table showing the quality assessment scores for the individual checklist items for each included study. (PDF 44 kb) [file 13047_2015_85_MOESM3_ESM.pdf]

Additional file 3

Quality assessment scores for individual checklist items

Reference	Reporting (max = 11)										External validity (max = 3)			Internal validity													Power (max = 5)	Total (max = 32)
														Bias (max = 7)							Confounding (max = 6)							
	**1**	**2**	**3**	**4**	**5**	**6**	**7**	**8**	**9**	**10**	**11**	**12**	**13**	**14**	**15**	**16**	**17**	**18**	**19**	**20**	**21**	**22**	**23**	**24**	**25**	**26**	**27**	
Allam, 2006 [21]	1	1	0	1	2	1	1	1	0	1	0	0	0	0	0	1	0	1	1	1	0	0	1	0	1	0	3	18
Batista *et al.*, 2011 [26]	1	0	1	1	1	0	0	1	0	0	0	0	0	0	0	1	0	0	1	0	1	1	0	0	0	0	0	9
Colen *et al.*, 2013 [27]	1	1	1	1	2	1	0	1	0	1	1	1	0	0	0	1	1	1	1	1	1	0	0	0	1	0	3	21
Dayer *et al.*, 2009 [28]	1	1	1	1	2	1	1	1	1	0	0	0	0	0	0	1	0	0	1	1	0	1	0	0	0	1	0	15
Hamilton *et al.*, 2005 [29]	1	1	1	1	1	1	1	1	1	0	1	1	0	0	0	1	0	0	1	0	1	1	0	0	0	1	0	16
Kim *et al.*, 2012 [22]	1	1	1	1	2	1	1	1	0	0	0	0	0	0	0	1	0	1	1	1	0	1	0	0	1	0	3	18
Laborde, 2005 [30]	1	1	1	1	2	1	0	1	1	0	1	1	0	0	0	1	0	0	1	0	0	1	0	0	0	1	0	15
Laborde, 2009 [31]	1	1	1	1	1	1	0	1	1	0	1	1	0	0	0	1	0	0	1	0	0	1	0	0	0	1	0	14
La Fontaine *et al.,* 2008 [32]	1	1	1	1	1	1	1	1	0	1	1	1	1	0	0	1	0	1	1	1	1	1	0	0	1	0	5	24
Lin *et al.*, 1996 [33]	1	0	1	1	2	1	1	1	0	0	0	0	0	0	0	1	0	0	1	1	0	1	0	0	0	0	0	12
Mueller *et al.*, 2003 [15]	1	1	1	1	2	1	1	1	1	1	0	0	0	0	0	1	1	1	1	1	1	1	1	1	1	1	5	27
